# Mycobacterium haemophilum: Emerging or Underdiagnosed in Brazil?

**DOI:** 10.3201/eid0811.020492

**Published:** 2002-11

**Authors:** Jorge Luiz Mello Sampaio, Venâncio Avancini Ferreira Alves, Sylvia Cardoso Leão, Vanda Dolabela de Magalhães, Marinês Dalla Valle Martino, Caio Marcio Figueiredo Mendes, Antonio Carlos de Oliveira Misiara, Kozue Miyashiro, Jacyr Pasternak, Eliana Rodrigues, Ronaldo Rozenbaum, Carlos Alberto Sant´Anna Filho, Sônia Regina Marques Teixeira, Adriano Cunha Xavier, Mauro Silvério Figueiredo, José Paulo Gagliardi Leite

**Affiliations:** * Fleury - Centers for Diagnostic Medicine, São Paulo, Brazil; † Federal University of São Paulo, São Paulo, Brazil; ‡ Oswaldo Cruz Hospital, São Paulo, Brazil; § Albert Einstein Hospital, São Paulo, Brazil; ¶ Sírio Libanês Hospital, São Paulo, Brazil; # Servidores do Estado Hospital, Rio de Janeiro, Brazil; ** Aliança Hospital, Salvador, Bahia, Brazil; †† Lâmina Laboratory, Rio de Janeiro, Brazil; ‡‡ Oswaldo Cruz Institute, Rio de Janeiro, Brazil

**To the Editor:** *Mycobacterium haemophilum* was first described in 1978 by Sompolinsky et al. (1) as the cause of cutaneous infections in a patient with Hodgkin disease. Since then, fewer than 100 cases have been reported worldwide, mostly among immunocompromised patients (2), although *M. haemophilum* infection has also been described in immunocompetent patients as the cause of cervical, submandibular, and perihilar lymphadenopathy in children and of pulmonary nodules in an adult (3–5). Cases have been reported from United States, Australia, Canada, France, Israel, and the United Kingdom, but to date no reports have originated in South America.

The most frequent clinical sign of *M. haemophilum* infection in adults is a skin or joint lesion. Less common sites for isolation of *M. haemophilum* include the respiratory tract, blood, bone marrow, bone, and central venous catheters (2,6). *M. haemophilum* is unique among *Mycobacterium* species owing to its special growth requirements: it grows best at 30°C and requires an iron supplement (hemin or ferric ammonium citrate).

We report here the characterization of three strains of *M. haemophilum* isolated from patients living in three states in two distinct regions of Brazil, Rio de Janeiro and São Paulo (southeast region) and Bahia (northeast region). The first strain was detected in Rio de Janeiro in December 2000 from a blood culture of a 67-year-old man who had received a kidney transplant in 1988 at the age of 55 years and was undergoing immunosuppressive treatment with prednisolone and mycophenolate mofetil. The second strain was detected in São Paulo in March 2001 in a 43-year-old HIV-seropositive man from a biopsied specimen of a nasal ulcer. A direct acid-fast stain showed many acid-fast bacilli. At time of diagnosis, the patient’s CD4+ cell count was 8/mm^3^ and his viral load was 290.000 copies/mL. The third isolate was detected in Bahia in a 30-year-old HIV-seropositive man who had osteomyelitis in an elbow. A direct acid-fast stain showed rare acid-fast bacilli.

The isolate from the Rio de Janeiro patient grew only in Myco/F Lytic media (Becton Dickinson Microbiology Systems, Sparks, MD) plus blood in primary isolation and subculture; it failed to grow on chocolate agar at 30°C after 6 weeks. The isolates from São Paulo and Bahia showed a slight growth in 12B media on primary isolation; this growth was likely supported by the iron provided by the biopsied tissue. Subcultures on chocolate agar showed good growth after 2–3 weeks at 30°C. The isolate did not grow on Middlebrook 7H10 agar without hemin and grew on the same media when supplemented with 60 μM of hemin. Both strains showed a negative catalase reaction.

The species of all isolates was identified through polymerase chain reaction amplification of the gene encoding for the 65-kDa heat shock protein, followed by restriction analysis with the enzymes *Bst*EII and *Hae*III as described by Telenti et al. (7), with minor modifications. The three isolates showed the same restriction pattern as that obtained for *M. haemophilum* American Type Culture Collection 29548 prototype strain. Isolates from Rio de Janeiro and São Paulo were also molecularly characterized as previously described by Roth et al. (8), corroborating *M. haemophilum* species identification.

To our knowledge, these *M. haemophilum* isolates are the first to be reported in Brazil. These three patients came from cities 429–962 km apart, demonstrating the dispersion of *M. haemophilum* infection in Brazil. Given the specific requirements of *M. haemophilum* for its growth in culture, our findings suggest that its true incidence in Brazil is greatly underestimated. Consequently, we strongly recommend that clinical laboratories in Brazil include an iron-supplemented medium, such as chocolate agar, incubated at 30ºC, for primary isolation of *Mycobacterium* spp in samples from selected patients.
